# Heparan sulfate proteoglycans undergo differential expression alterations in left sided colorectal cancer, depending on their metastatic character

**DOI:** 10.1186/s12885-018-4597-x

**Published:** 2018-06-25

**Authors:** Ainara Crespo, Olivia García-Suárez, Iván Fernández-Vega, María Pilar Solis-Hernandez, Beatriz García, Sonia Castañón, Luis M. Quirós

**Affiliations:** 1 Department of Biotechnology, Neiker-Tecnalia Arkaute, 01080 Vitoria-Gasteiz, Spain; 2 Instituto Universitario Fernández-Vega, and Department of Morphology and Cell Biology, University of Oviedo, 33006 Oviedo, Spain; 3 Instituto Universitario Fernández-Vega, and Department of Pathology, Hospital Universitario Central de Asturias, Oviedo, 33006 Spain; 4 Department of Surgery and Medical-surgical Specialties, University of Oviedo, 33006 Oviedo, Spain; 5 Department of Medical Oncology, Hospital Universitario Central de Asturias, Oviedo, Spain; 6 Instituto Universitario Fernández-Vega, and Department of Functional Biology, University of Oviedo, 33006 Oviedo, Spain

**Keywords:** Colorectal cancer, Heparan sulfate, Proteoglycan, Glycosaminoglycan, Chondroitin sulfate

## Abstract

**Background:**

Heparan sulfate proteoglycans (HSPGs) are complex molecules which play a role in the invasion and growth and metastatic properties of cancerous cells. In this work we analyze changes in the patterns of expression of HSPGs in left sided colorectal cancer (LSCRC), both metastatic and non-metastatic, and the results are also compared with those previously obtained for right sided tumors (RSCRCs).

**Methods:**

Eighteen LSCRCs were studied using qPCR to analyze the expression of both the proteoglycan core proteins and the enzymes involved in heparan sulfate chain biosynthesis. Certain HSPGs also carry chondroitin sulfate chains and so we also studied the genes involved in its biosynthesis. The expression of certain genes that showed significant expression differences were also analysed using immunohistochemical techniques.

**Results:**

Changes in proteoglycan core proteins were dependent on their location, and the main differences between metastatic and non-metastatic tumors affected cell-surface glypicans, while other molecules were quite similar. Glypicans were also responsible for the main differences between RS- and LS- malignances. Regarding the biosynthesis of heparan sulfate chains, differential alterations in transcription depending on the presence or not of metastasis affected genes involved in the modification of uronic acid (epimerization and 2-O sulfation), and some isoforms responsible for sulfation of glucosamine (*NDST1*, *HS6ST1)*. Moreover, in RSCRCs differences were preferentially found in the expression of genes involved in C6 and C3 sulfation of glucosamine, but not in NDSTs or SULFs. Finally, synthesis of chondroitin sulfate showed some alterations, which affected various steps, including polimerization and the modification of chains, but the main variations dependent on the presence of metastases were epimerization and 6C sulfation; however, when compared with RSCRCs, the essential divergences affected polymerization of the chains and the 6C sulfation of the galactosamine residue.

**Conclusions:**

We evidenced alterations in the expression of HSPGs, including the expression of cell surface core proteins, many glycosiltransferases and some enzymes that modify the GAG chains in LSCRCs, but this was dependent on the metastatic nature of the tumor. Some of these alterations are shared with RSCRCs, while others, focused on specific gene groups, are dependent on tumor localization.

**Electronic supplementary material:**

The online version of this article (10.1186/s12885-018-4597-x) contains supplementary material, which is available to authorized users.

## Background

Colorectal cancer (CRC) is a heterogeneous disease and, despite their similar histological aspect, there are substantial differences between left- and right sided CRCs (LSCRCs and RSCRCs), including their etiology, response to screening tests, the stage at which they are diagnosed, and their effect on mortality [1]. At the molecular level, differences in the expression of different biomarkers have been described between LS- and RSCRCs.

Interestingly, several of these markers are related to heparan sulfate proteoglycans (HSPGs) including: p53, related to the regulation of genes as SULF2 and heparanase [2, 3]; MUC1, involved in cell-cell dissociation and invasiveness, in cooperation with HSPGs [4]; the Wnt/β-catenin pathway, regulated by glypican-3 and -4 [5, 6]; cytokines like VEGF, EGF and TGF-beta and other markers, which bind to heparan sulfate (HS) chains, which regulate their activity [7–9].

HSPGs comprise a specific small group of glycoconjugates composed of various core proteins post-translationally modified with HS glycosaminoglycan (GAG) chains. HS is a complex linear anionic polysaccharide whose synthesis occurs mainly in the Golgi apparatus. It is initiated by the formation of a tetrasaccharide linkage on the protein core after which the HS chain is elongated by the addition of alternating D-glucuronic acid (GlcA) and N-acetyl-D-glucosamine (GlcNAc) residues. Subsequently, a series of different enzymatic reactions act in an orderly manner on specific regions of the molecule, including N-sulfation, epimerization and various O-sulfations. This gives rise to highly sulfated regions (NS domains), which alternate with nonsulfated (NA domains) and mixed (NA/NS) domains [10, 11].

Since function ultimately relies on the fine structure of the chains, cells exercise accurate control over HSPG composition and sequence, which results in these molecules varying depending on factors like cell type and development stage, as well as due to pathological processes. The binding sites for a great variety of ligands, such as cytokines, chemokines, growth factors, enzymes and extracellular matrix (ECM) proteins [11, 12] are defined by specific sets of variably modified disaccharides, usually within the NS domains. These networks of complex interactions at the molecular level mean that HSPGs participate actively in the control of many normal physiological functions [10–13]. Because of these interactions, HS is also involved in many pathologies, including inflammation, amyloid diseases, infectious diseases and cancer [14]. Given that function is dictated by the fine structure of the chains, the detailed analysis the full set of changes in the expression of HSPG core proteins and HS biosynthetic enzymes in cancer pathologies is of great interest, as is a detailed consideration of the effect of these particular signatures on invasion and metastasis.

Up- or downregulation of genes involved in the biosynthesis of HSPGs have been reported in many cancerous cells [15]. In the case of CRCs, various alterations have been described, for example, those relating to specific syndecans [16], the relative amounts and structure of glycosaminoglycans [17, 18] and the expression levels of certain enzymes involved in HS saccharidic chain structure [19, 20]. However, many of the previous studies reported in the literature analyze this pathology in a general way even though, as indicated above, CRC is a heterogeneous disease with respect to the anatomical location of the tumor.

We recently published the results of a study focused on RSCRCs [21] where, since the presence or absence of metastases in lymph nodes is a key predictor of progression, we subdivided the tumors into two groups according to this important feature. We found that the number of genes affected was higher in non-metastatic tumors, with around 40% of all genes analyzed being involved, and that most of the genes whose expression was altered in metastatic malignances also showed altered expression in non-metastatic tumors. Additionally, the PGs located at the cell surface showed significant differences in expression depending on the presence or absence of metastases, while alterations of those located in the ECM or within the cell were very similar in both tumor types. HS chains seemed to experience far more limited changes in metastatic CRCs than in non-metastatic tumors, while chondroitin sulfate (CS) chains, which are also carried by some HSPGs, were strongly affected, albeit differently in the two tumor groups [21]. In this current paper, we have investigated the expression patterns of all the genes involved in HSPG biosynthesis in LSCRCs, compared with healthy tissues from the same patients. As in the previous study focusing on RSCRCs, the tumors were subdivided into two groups according to presence or absence of metastases in lymph nodes, and the study included genes coding for HSPG core protein and GAG chain synthesis and modification. The aim of the work is to increase our knowledge of structural alterations of HSPGs in LSCRCs, comparing the data to that previously obtained for RSCRCs, in an attempt to define biomarkers which are different in metastatic and non-metastatic tumors which could be useful in the future to develop new chemical biology approaches to retard tumor progression by modulating deregulated biosynthetic pathways.

## Methods

### Tissue samples

We analyzed a cohort of 36 snap frozen colon samples, obtained from the Tumor Bank at the Instituto de Oncología Asturias (IUOPA, Asturias, Spain). 18 of the samples were from LSCRCs, while the remaining 18 were used as control and were from the corresponding surrounding healthy tissue from the same patients. Diagnoses were carried out according to the World Health Organization (WHO) criteria using hematoxylin-eosin-stained slides and the snap frozen tissues were stored at − 80 °C prior to isolation of the RNA. Informed written consent of all the patients was obtained, and the study was approved by the Ethics Committee on Clinical Investigation of the Hospital Universitario Central de Asturias.

### Total RNA isolation and cDNA synthesis

Tissue fragments (20–30 mg) were homogenized using a polytron PT 2100 (Kinematica Inc.; Bohemia, NY), and RNA was isolated using the RNeasy kit (Qiagen, Hilden, Germany), and processed as previously described [21]. cDNA synthesis was carried out using the High Capacity cDNA Transcription Kit (Applied Biosystems, Foster City, CA, USA). The reactions were performed and the products cleaned and stored as previously described [21].

### Quantitative real-time polymerase chain reactions (qRT-PCR)

qRT-PCR reactions, and analysis of amplimer products were carried out accordingly to methods already detailed [21]. Actin was used as the control gene to normalize individual gene expression.

### Data analysis

Statistical analysis of the data and expression of the values of differential transcription were performed as previously described [21].

The overall survival (OS) and cumulative probability analyses were performed using the Kaplan–Meier method and the survival curves were compared by the log Rank test, using IBM® SPSS® Statistics V.21.

### Tissue microarray construction and immunohistochemistry

Representative tumor regions were identified in each sample and selected to make a tissue microarray containing three tissue cores from each sample of LSCRC. After 5 min at 60 °C the tissue microarray blocks were cut in 4 μm thick sections, ready for immunohistochemical techniques. Tissue sections were treated, prepared and immunostained as previously described [21]. For the detection of chondroitin 6-sulfotransferase-2, syndecan 1, CD117, NDST1 and glypican-4, sections were heated in high pH Envision FLEX target retrieval solution at 65 °C for 20 min and then incubated for 20 min at room temperature in the same solution. To detect perlecan, CS, HS2ST1, UST, CS, CS4ST, the same procedure was followed except that the final step was omitted and the sections were instead incubated overnight at 4 °C in a humid chamber with primary antibodies. The antibodies and the dilution are detailed in the Additional file 1.

After the first incubation, sections were rinsed in the same buffer, and incubated with the following secondary antibodies; anti-rabbit, anti-mouse EnVision system-labelled polymer (DakoCytomation) and anti-goat diluted 1:100 (Santa Cruz Biotechnology) for 1 h at room temperature. Finally, the sections were washed and the immunoreaction visualized using 3–3’DAB as a chromogen. The sections were studied and photographed under a light microscope (Eclipse 80i; Nikon Corporation, Tokyo, Japan).

## Results

### Analysis of differential gene expression

Almost all the genes known to be involved in the various steps defined in the biosynthesis of HSPGs in LSCRCs were investigated in this work. The tumor samples used in the present study, all of them non-mucinous, were obtained from the Tumor Bank of IUOPA. Diagnosis was made by staining with hematoxylin and eosin according to the criteria of the World Health Organization (WHO).

Applying the TNM classification, all tumors were at the T3 stage (muscularis propria affected) and were classified into two groups depending on the presence (at least N1) or absence (N0) of lymph node metastases, which resulted in 10 samples being included in the first group and 8 in the second. Non-metastatic tumor samples, all of them of stage IIA, came from 7 male patients and 3 female patients, all of them between the ages of 60 and 81 years. Among the metastatic samples, i.e. stage IV, 5 were from male patients and 3 from females, and ages ranged from 60 to 88 years.

We used qRT-PCR to perform a quantitative analysis of mRNA expression. In many of the genes where we were able to detect differences between normal tissues and tumors, we complemented the studies using immunohistochemistry.

### Differential expression of genes encoding core proteins carrying HS chains

**Fig. 1:**
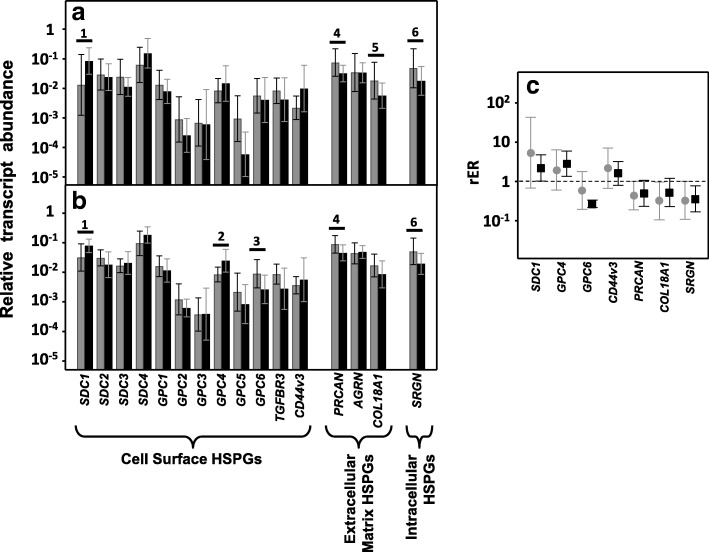
Differential transcription of genes encoding HSPGs. **a**, **b**, Relative transcript abundance of mRNAs for healthy tissues (gray bars) and tumors (black bars). Genes that show significant differences in their transcription levels are highlighted. **a**, Non-metastatic LSCRCs. 1: syndecan-1 (*p* < 0.05); 4: perlecan (*p* < 0.05); 5: collagen XVIII (*p* < 0.05); serglycin (*p* < 0.05). **b**, Metastatic LSCRCs. 1: syndecan-1 (*p* < 0.035); 2: glypican-4 (*p* < 0.05); 3: glypican-6 (*p* < 0.05); 4: perlecan (*p* < 0.05); 8: serglycin (*p* < 0.05). **c**, Relative expression ratio of genes that show statistically significant differences in expression in non-metastatic (●) or metastatic (■) CRCs. Values on the Y axis are on a logarithmic scale and the spreads represent the standard deviations

Only 13 genes encode HSPGs core proteins. The great majority of cell surface HSPGs are related to two gene families, the syndecans and the glypicans, which comprise 4 and 6 isoforms, respectively (SDC1–4 and GPC1–6); the three remaining molecules are arranged in the extracellular matrix and are perlecan (PRCAN), agrin (AGRN) and collagen type XVIII (COL18A1) [22]. Within the group of syndecans, no significant differences in transcript levels of isoforms 2, 3 and 4 were detected, irrespective of the presence or absence of lymph node metastasis (Fig. 1a and b). However, 80% of non-metastatic tumors (*p* < 0.05) exhibited an overexpression of syndecan 1 mRNA, the average being a more than 3-fold increase, as did 87% of metastatic tumors (*p* < 0.05), although in this case the mean increase was around 70% (Fig. 1c).

**Fig. 2:**
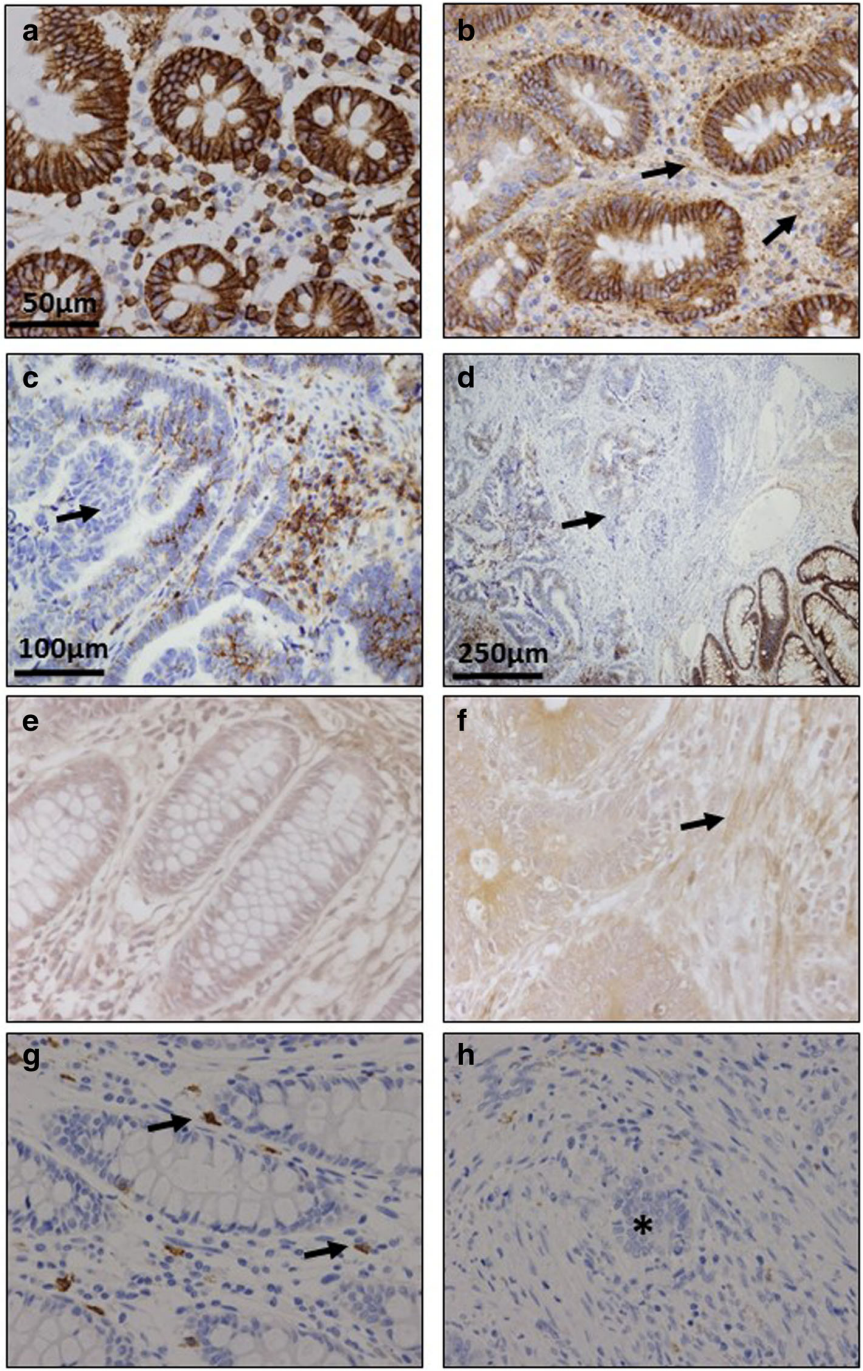
Immunolocalization of cell HSPGs. **a**-**d**, Histological localization of syndecan-1 expression. **a**, Normal mucosa showing intense staining associated with the cell membrane. Plasma cells in lamina propria are also stained positively. **b**, Non-metastatic tumor displaying decreased immunoreactivity and weak staining in ECM (arrows). Plasma cells in lamina propria are also positively stained; magnification 40X. **c**, Metastatic tumor and (**d**), Transition area between normal colon mucosa and tumor; arrows indicate the loss of staining in the tumor region; magnification 20X and 10X respectively. **e**-**f** Immunolocalization of glypican-4 expression in normal mucosa **(e)** and metastatic tumor **(f**); In the healthy tissue weak immunomarking is observed in crypts and matrix which is stronger in the tumor tissue, as it also is in the fibrous tissue (arrow); magnification 40X. (**g-h**), Immunolocalization of mast cells using CD117 antibody. Localization of mast cells in normal colon mucosa (**g**) and tumor (**h**). Arrows indicate the staining of mast cells in the normal mucosa. The asterisks show the tumor area, where there is no detectable staining. Magnification 40X

Immunohistochemistry was used to investigate changes in syndecan 1, using monoclonal anti-SDC1 (Fig. 2a-d). Non-metastatic tumors demonstrated considerable reactivity but, interestingly, lower than those detectable in healthy tissue (Fig. 2a); In addition, there were differences in the localization of the immunostaining: in the healthy tissue it appeared to be associated with the cellular membranes, while in the tumor tissue it was also detected in the extracellular matrix, probably due to shedding of cell membrane-bound proteoglycans (Fig. 2b). In contrast, and surprisingly, the metastatic tumors showed a dramatic decrease in staining, indeed largely non-existant, while labeling was very clear in the adjacent normal mucosa (Fig. 2c and d).

Analysis of the expression of the different glypicans revealed the presence of transcripts for the 6 different isoforms, although their levels varied widely depending on the particular isoform examined. The qRT-PCR results were unable to detect significant changes in the levels of transcripts in non-metastatic tumors (Fig. 1a), while in metastatic LSCRCs *GPC4* was overexpressed in 87% of cases (*p* < 0.05), with an approximately 3-fold increase (Fig. 1b and c). These results were also observed by immunohistochemistry, where *GPC4* staining was found to be elevated in tumor tissue (Fig. 2e and f). In metastatic tumors, a significant downregulation of *GPC6* was also detected, its levels being reduced in 87% of the cases analyzed (*p* < 0.05), with values being around 26% of those determined in healthy tissues (Fig. 1b and c).

Two other *part time* HSPGs may be present in the cell membrane in addition to syndecans and glypicans: betaglycan (*TGFBR3)* and CD44v3 [22]. The results from the qRT-PCR analysis identified transcripts of these genes in both metastatic and non-metastatic tumors as well as in normal tissue (Fig. 1a and b), although there were no significant differences in their transcript levels (Fig. 1c).

qRT-PCR analysis of serglycin, a cell-associated PG which is intracellular [23], unlike other HSPGs, showed decreased levels, around 35% of the values obtained for healthy tissues, independent of the metastatic nature of the tumor (Fig. 1), and affecting 70–75% of the samples analyzed (*p* < 0.05). Since serglycin is principally located in mast cell secretory granules [23], we were prompted to attempt to detect these cells through immunohistochemical studies using the antibody CD117. The results highlighted a dramatic reduction in the population of mast cells in tumoral compared to non-tumoral colon mucosa (Fig. 2g and h).

**Fig. 3:**
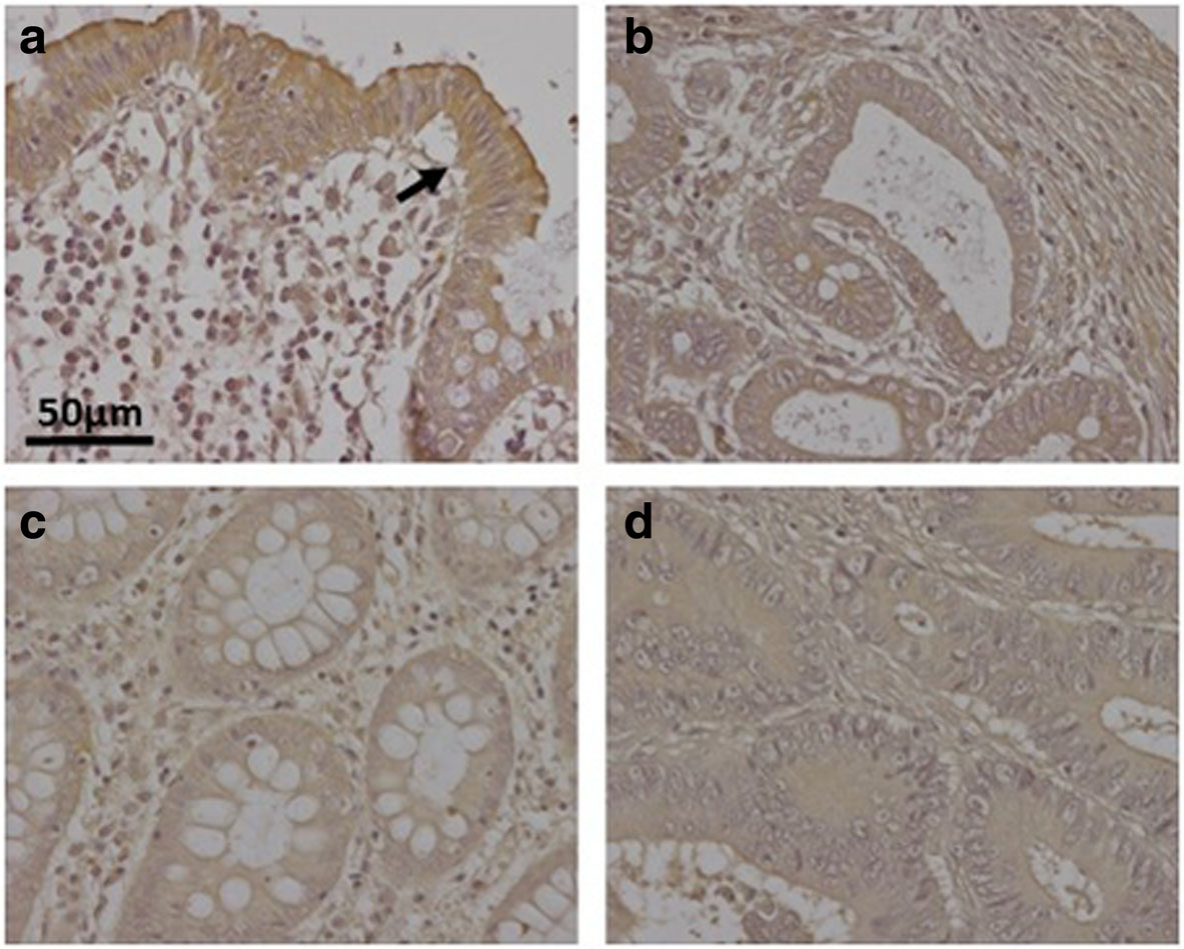
Immunolocalization of ECM HSPGs. **a-b** Immunolocalization of perlecan expression. **a**, Normal mucosa showing positive staining in the cell cytoplasm and in the basement membranes, magnification 40X. **b**, Non-metastatic tumors; magnification 40X. **c-d**, Immunolocalization of collagen XVIII expression. **c**, Normal mucosa and (**d**), Non-metastatic LSCRCs showing no positive immunoreactivity; Magnification 40X

As regards extracellular matrix PGs, no significant differences in levels of agrin were found (Fig. 1a and b), although transcription levels of perlecan decreased in about 75% of tumor samples (*p* < 0.05), with values being around 60% lower than those obtained for healthy tissues (Fig. 1c). These results were also observed by immunohistochemistry, where perlecan displayed faint staining in normal tissues, while in metastatic and non-metastatic LSCRCs staining in the tumor stroma was weaker still (Fig. 3a and b). Moreover, a significant underexpression of collagen XVIII in non-metastatic tumors was detected (Fig. 1a and b), which was 70% lower than that of healthy tissue (*p* < 0.05) (Fig. 1c), and observed in 70% of the LSCRCs analyzed. In the case of metastatic tumors, the differences observed approached significance (*p* = 0.07), and transcription values were around 50% lower in 70% of samples (Fig. 1c). However, the expression of the protein could not be detected via immunohistochemistry in either healthy tissue or tumor samples (Fig. 3c and d).

### Differential expression of genes encoding glycosyltransferases involved in common linkage region sequence and GAG chain synthesis

**Fig. 4:**
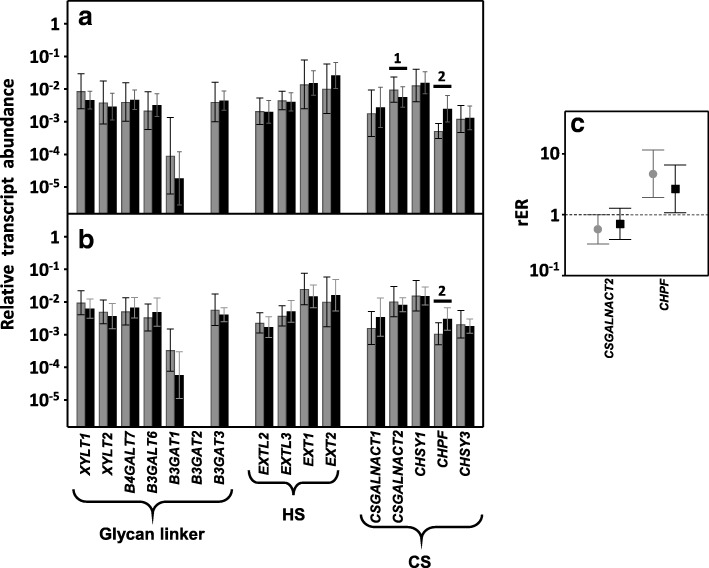
Transcription of genes encoding the glycosyltransferases involved in the biosynthesis of HS and CS chains. **a**, **b**, Relative transcript abundance of mRNAs for healthy tissues (gray bars) and tumors (black bars). Genes that display significant differences in their transcription levels are highlighted. **a**, Non-metastatic LSCRCs. 1: N-acetylgalactosaminyltransferase 2 (*p* < 0.05); 2: chondroitin polymerizing factor (*p* < 0.05). **b**, Metastatic LSCRCs. 2: chondroitin polymerizing factor (*p* < 0.05). **c**, Relative expression ratio of genes that showed statistically significant differences in expression in non-metastatic (●) and metastatic (■) LSCRCs. Values on the Y axis are on a logarithmic scale and the spreads represent the standard deviations

The synthesis of HS and CS chains is dependent on the cooperation of a number of biosynthetic enzymes in the Golgi. The initial step involves a tetrasaccharide glycan linker being synthesized on a cognate serine residue of the proteoglycan core, whose sequence is integrated by xylose-galactose-galactose-GlcA [24, 25]. The genes encoding the glycosyltransferases (GTs) involved in this process are: *XYLT1* and *XYLT2*, which ensures the initial transfer of xylose residue; *B4GALT7* and *B3GALT6,* responsible for the sequential addition of the two residues of galactose; and *B3GAT1*, *B3GAT2* and *B3GAT3*, each of which encodes the enzymes responsible for the transference of GlcA [26]. Transcripts for all these genes except *B3GAT2* were detected both in healthy tissues and in LSCRCs, but none of them showed significant differences in their expression levels (Fig. 4a and b).

The subsequent chain extension of HS involves the transfer of a GlcNAc residue, followed by the sequential addition of alternate GlcA and GlcNAc residues, which results in a non-branched polymer. The mRNA levels of none of the GTs involved in the synthesis of the HS chains appeared modified in CRCs, including the enzymes involved in the transference of the first glucosamine residue (*EXTL2*, *EXTL3)* and those responsible for the subsequent polymerization (*EXT1*, *EXT2)* (Fig. 4a and b).

The linker is shared by HS and CS chains, and the addition of an N-acetyl-D-galactosamine (GalNAc) rather than GlcNAc directs the pathway towards the biosynthesis of CS. In this case, chain extension takes place through the sequential addition of alternate GlcA and GalNAc residues [23, 27]. One of the genes involved in the transference of the first GalNAc residue, *CSGALNACT2* appeared downregulated by around 50% in 70% of non-metastatic LSCRCs (*p* < 0.05), whereas in metastatic tumors the differences were not statistically significant (*p* = 0.32). No differences in the transcription of the chondroitin synthases *CHSY1* and *CHSY3* were detected, however, the chondroitin polymerizing factor *CHPF* was overexpressed in all the tumoral samples analyzed, both metastatic and non-metastatic (*p* < 0.05) (Fig. 4c).

### Differential expression of genes involved in HS chain modification

**Fig. 5:**
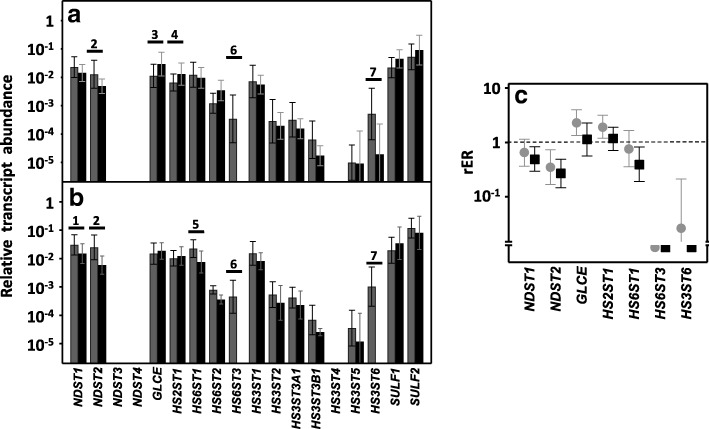
Differential transcription of genes involved in the modification of HS chains. **a**, **b**, Relative transcript abundance of mRNAs for healthy tissues (gray bars) and tumors (black bars). Genes that display significant differences in their transcription levels are highlighted. **a**, Non-metastatic LSCRCs. 2: N-deacetylase/N-sulfotransferase 2 (*p* < 0.05); 3: C5-GlcA epimerase (*p* < 0.05); 4: 2-O-sulfotransferase (*p* < 0.05); 6: 6-O-sulfotransferase 3 (*p* < 0.001); 7: 3-O-sulfotransferase 6 (*p* < 0.05). **b**, Metastatic LSCRCs. 1: N-deacetylase/N-sulfotransferase 1 (*p* < 0.05); 2: N-deacetylase/N-sulfotransferase 2 (*p* < 0.01); 5: 6-O-sulfotransferase 1 (*p* < 0.05); 6: 6-O-sulfotransferase 3 (*p* < 0.001); 7: 3-O-sulfotransferase 6 (*p* < 0.001). **c**, Relative expression ratio of genes that show statistically significant differences in expression in non-metastatic (●) and metastatic (■) LSCRCs. Values on the Y axis are on a logarithmic scale and the spreads represent the standard deviations

**Fig. 6:**
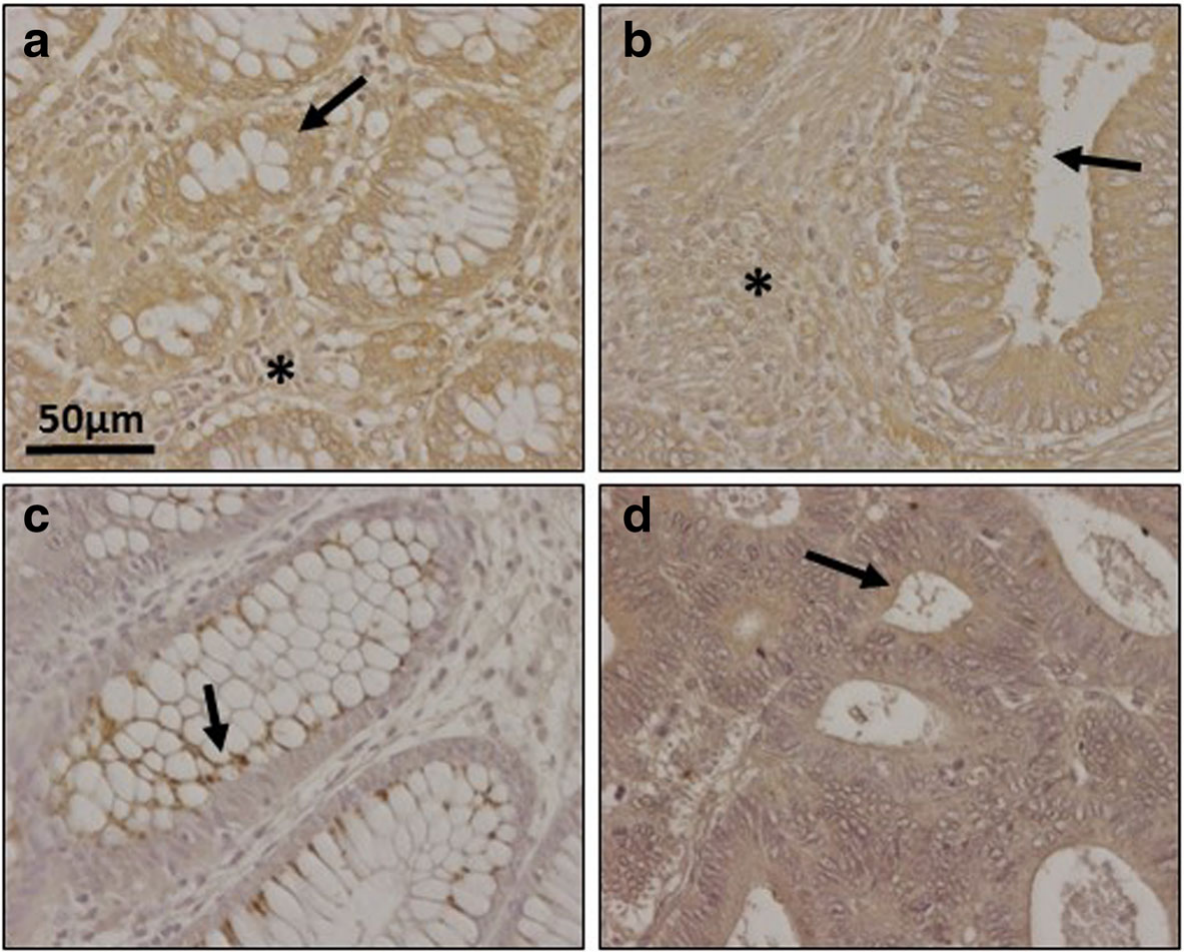
Immunolocalization of enzymes involved in the modification of HS chains. **a**-**b** Immunolocalization of N-deacetylase/N-sulfotransferase-1. **a**, Normal mucosa showing positive weak staining mainly in crypts (arrow) and in the extracellular matrix (asterisk). **b**, Metastatic tumor showing a decrease in staining in both locations; magnification 40X. **c**-**d**, Immunolocalization of HS 2-O-sulfotransferase. **c**, Normal mucosa showing faint staining in the crypts, with a vesicular punctate pattern (arrow), and in some basal cells, no marking in the matrix (**d**), Non-metastatic LSCRCs with a slightly higher immunoreaction; magnification 40X

As the HS chain polymerizes, a series of modifications occur, the first being the removal of acetyl groups from GlcNAc residues. This is followed by the sulfation of the amino group, which is catalyzed by four different isoforms of N-deacetylase/N-sulfotransferases: NDST1, NDST2, NDST3 and NDST4 [12, 13]. Transcripts were found for only two of these isoforms, *NDST1* and *− 2*, but these were quantified in all healthy and tumoral tissues. *NDST3* transcripts, on the other hand, were detected in only a percentage of tumors (less than 40%), while *NDST4* was undetectable in most samples (Fig. 5a and b). *NDST2* appeared downregulated by 65% in non-metastatic LSCRCs (*p* < 0.05) and by 75% in metastatic (*p* < 0.01), in 80 and 100% of the respective samples (Fig. 5c). In metastatic tumors, *NDST1* transcription levels were around 60% lower than controls in 75% of cases (*p* < 0.05) (Fig. 5), while in non-metastatic samples, the decrease was only around 40%, in 60% of the cases, though it was not statistically significant (*p* = 0.07) (Fig. 5). Changes in *NDST1* were also analyzed by immunohistochemistry, showing weak staining in healthy tissues, mainly in crypts and in the extracellular matrix, which was even weaker in tumoral tissue (Fig. 6a and b).

The next modifications of the HS chain include two reactions involving the GlcA residue: epimerization into IdoA, catalyzed by the enzyme C5-GlcA epimerase (encoded by *GLCE*), and the addition of sulfate groups at C2, catalyzed by the enzyme HS 2-O-sulfotransferase (*HS2ST1)*. In non-metastatic LSCRCs the two genes experienced an upregulation of about 2.5 and 2 fold respectively (*p* < 0.05 in each case) (Fig. 5), affecting 80% of samples. Moreover, there was a strong positive correlation between the levels of upregulation of the two genes for each individual sample (r Spearman 0.78). Changes in HS 2-O-sulfotransferase were also analyzed by immunohistochemistry, where a positive immunoreactivity was observed for certain basal cells, as well as weak staining in Lieberkhün crypts, with a vesicular dotted pattern; the labelling was slightly increased in tumoral tissues (Fig. 6c and d).

With regard to the genes responsible for the sulfation in C6 of glucosamine, in the healthy tissue the existence of transcripts of the three isoforms was demonstrated. In contrast, isoform 3 was not detected in any of the tumor samples, and isoform 1 only appeared in 80% of the metastatic tumors, where it was downregulated around 70% (*p* < 0.05) (Fig. 5).

The addition of a sulfate group at C3 of glucosamine is the final step in the biosynthesis of HS chains in the Golgi, catalyzed by HS 3-O-sulfotransferase isoforms 1–6 (HS3ST1, HS3ST2, HS3ST3A1, HS3ST3B1, HS3ST4, HS3ST5 and HS3ST6) [10, 11]. No transcripts were detected for some of the genes and, for those that were, levels varied considerably depending on each specific isoform. Only HS3ST6 showed statistically significant differences, with levels being 97% lower on average in non-metastatic tumors than controls (*p* < 0.05), although there was great variability between samples, and it was undetectable in metastatic tumors (*p* < 0.001) (Fig. 5).

Finally, HS patterning modifications occur at the cell surface, carried out by two cell surface sulfatases, SULF1 and SULF2, which remove glucosamine-6S groups from specific regions [10, 11]. None of these genes displayed any alterations in their transcription levels in LSCRCs (Fig. 5).

**Fig. 7:**
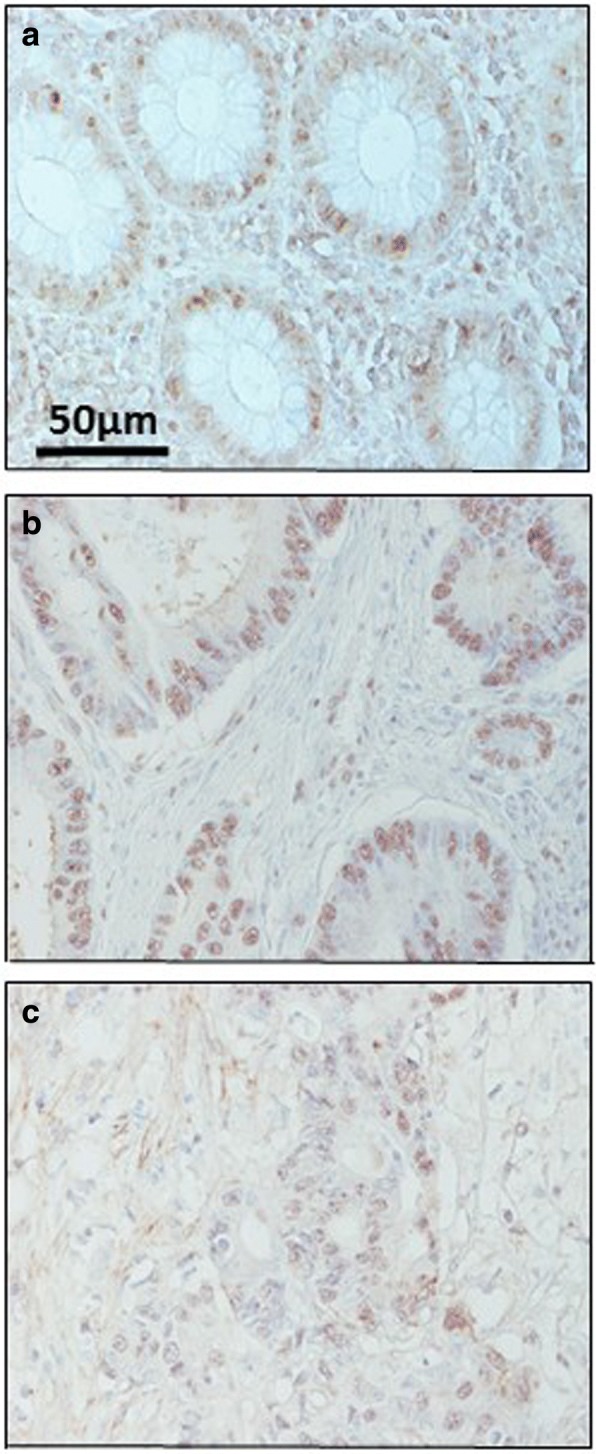
Immunolocalization of sulfated domains of HS chains using anti-HS 10E4 in colon mucosa and adenocarcinomas. **a**, Normal mucosa showing varying positive cytoplasmic (mostly perinuclear) and nuclear staining in both absorptive and goblet cells. (**b**, **c**), Colon adenocarcinomas showing variable degrees of highly sulfated domains of HS chains. A predominance of nuclear staining was noted, varying in degree of intensity from moderate (**b**) to negative or focally weak nuclear staining (**c**). Magnification 40X

However, since the results did demonstrate changes in the transcription levels of several enzymes involved in the modification of HS chains, we deduced that the structure of HS chains could also be altered. Consequently, we analyzed the distribution of HS molecules by immunohistochemistry using the specific antibody 10E4 which is able to recognize N-sulfated epitopes. Normal mucosa displayed positive cytoplasmic and nuclear staining in both absorptive and goblet cells, while in tumors, changes in the distribution and intensity of the immunostaining was detected (Fig. 7). LSCRCs showed variable degrees of sulfated HS chains, with a predominance of nuclear staining, which varied in its degrees of intensity (Fig. 7).

### Differential expression of genes involved in CS chain modification

**Fig. 8:**
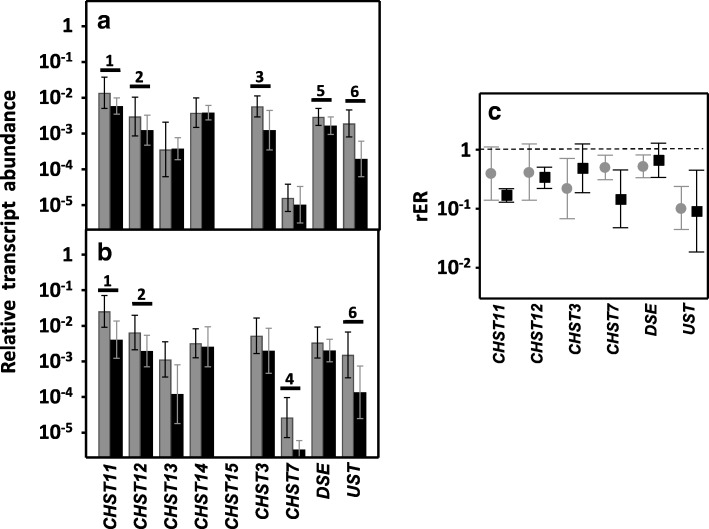
Differential transcription of genes involved in the modification of CS chains. **a**, **b**, Relative transcript abundance of mRNAs for healthy tissue (gray bars) and tumors (black bars. Genes that display significant differences in their transcription levels are highlighted. **a**, Non-metastatic LSCRCs. 1: chondroitin 4 sulfotransferase 1 (*p* < 0.05); 2: chondroitin 4 sulfotransferase 2 (*p* < 0.05); 3: chondroitin 6 sulfotransferase 1 (*p* < 0.05); 5: dermatan sulfate epimerase (*p* < 0.05); 6: uronyl-2-sulfotransferase (*p* < 0.01). **b**, Metastatic LSCRCs. 1: chondroitin 4 sulfotransferase 1 (*p* < 0.05); 2: chondroitin 4 sulfotransferase 2 (*p* < 0.05); 4: chondroitin 6 sulfotransferase 2 (*p* < 0.01); 6: uronyl-2-sulfotransferase (*p* = 0.012). **c**, Relative expression ratio of genes that show statistically significant differences in expression in non-metastatic (●) and metastatic (■) LSCRCs. Values on the Y axis are on a logarithmic scale and the spreads represent the standard deviations

**Fig. 9:**
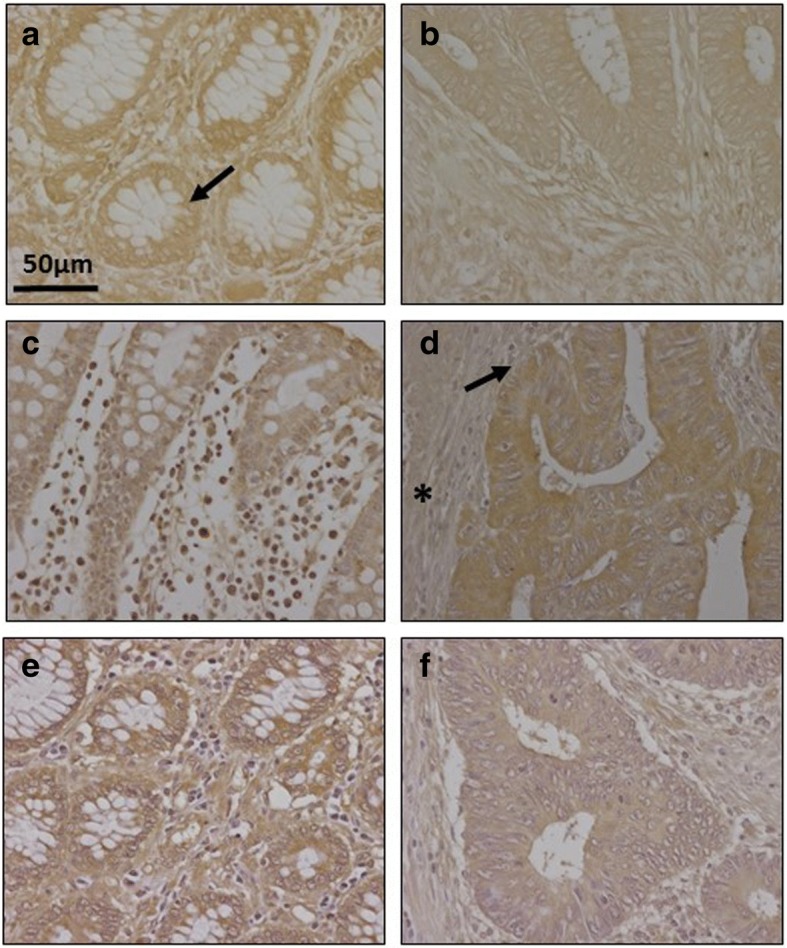
Immunolocalization of enzymes involved in the modification of CS chains. **a-b** Immunolocalization of chondroitin 4 sulfotransferase 2. **a**, Normal mucosa showing positive staining in crypts (arrow). **b**, Metastatic tumor showing a decrease in staining; magnification 40X. **c-d**, Immunolocalization of chondroitin 6 sulfotransferase 1. **c**, Normal mucosa, magnification 40X. **d**, Transition area between normal colon mucosa and tumor; the healthy tissue appears unmarked (asterisks), while the tumor shows intense immunoreactivity in the crypts (arrow), magnification 40X. **e-f**, Immunolocalization of uronyl-2-sulfotransferase. **e**, normal mucosa, (**f**), tumor, magnification 40X

Up to four different reactions can be involved in the modification of CS chains: GalNAc residues can be sulfated at C4 or C6, and IdoA residues can be epimerized at C5 or sulfated at C2 [24, 26]. Sulfation at C4 of GalNAc residues is catalyzed by four different enzymes, encoded by the genes *CHST11*–*14*, and the transcription of two of these genes appeared downregulated in LSCRCs. *CHST11* levels dropped by more than 60% in non-metastatic (*p* < 0.05) and around 85% in metastatic tumors (*p* < 0.01), affecting 85 and 100% of samples respectively (Fig. 8). *CHST12* was also downregulated in both types of tumor, with transcription levels decreasing about 60% in non-metastatic and 70% metastatic LSCRCs (*p* < 0.05), in 80 and 90% of the respective samples (Fig. 8). No statistically significant alterations were detected for the isoforms encoded by *CHST13* and *CHST14*. CHST12 protein expression was also analyzed by immunohistochemistry, showing healthy tissue immunoreaction in crypts and ECM, while staining was weaker in tumoral tissue (Fig. 9a and b).

Sulfation at C6 of GalNAc is catalyzed by three distinct enzymes, encoded by the genes *CHST15*, *CHST3* and *CHST7* [24, 26]. No transcripts for *CHST15* were detected in any of the samples studied; *CHST3* was downregulated about 80% in non-metastatic tumors (*p* < 0.05) in 80% of the samples analyzed, while *CHST7* decreased an average of more than 85% (*p* < 0.01) in all the metastatic samples (Fig. 8). Changes in CHST3 were also evaluated immunohistochemically but, interestingly, in this case the results showed an overexpression of the protein, with healthy tissue remaining unstained while tumoral tissue showed intense immunoreactivity in crypts (Fig. 9c and d).

Transcription levels of the two enzymes involved in the modification reactions of uronic acid residues were also significantly altered. DSE decreased by between around 50 and 80% in non-metastatic patients (*p* < 0.05), the differences in metastatic tumors were not, however, significant (Fig. 8). There was also a 10 fold decrease in *UST* in tumors, with all non-metastatic samples being affected, and 80% of metastatic (Fig. 8). The decrease was also determined at the protein level by immunohistochemistry, which revealed decreased staining in tumor samples (Fig. 9e and f).

**Fig. 10:**
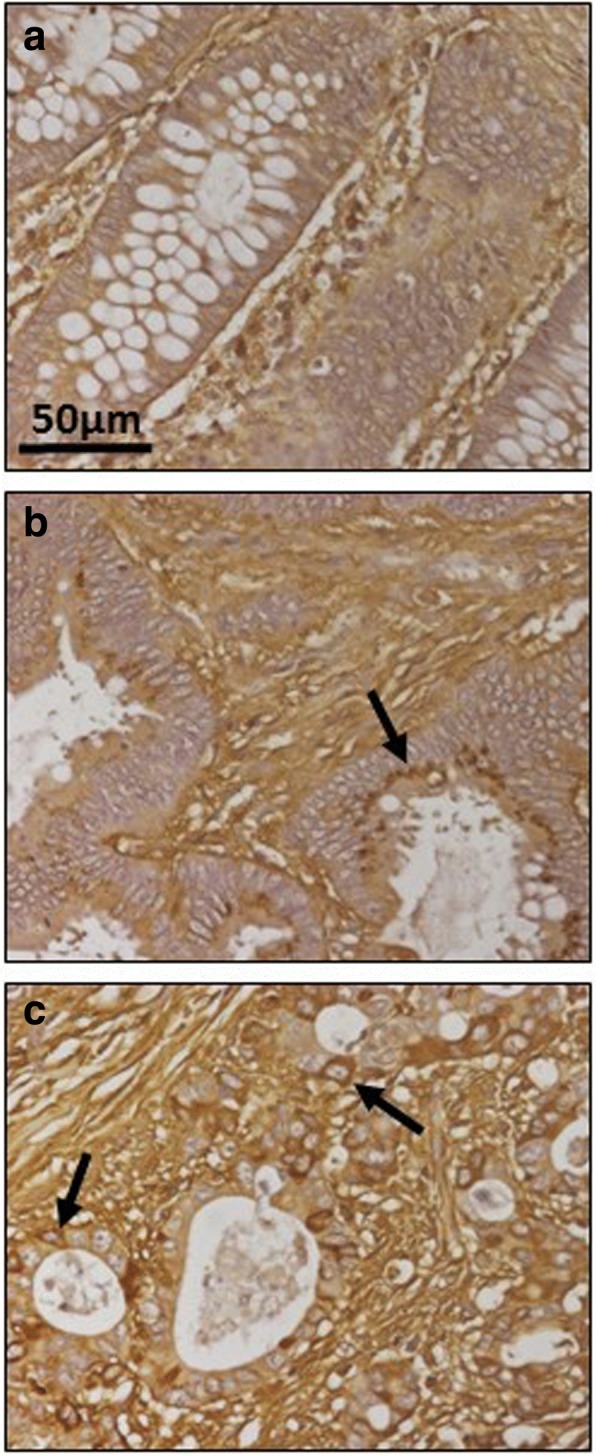
Immunolocalization of CS. **a**, Normal mucosa showing preferential staining in the stroma. **b**, Non-metastatic tumor, showing increased labelling, associated with the fibrous elements of the matrix, and also appearing on the crests with spotted staining in the cytoplasm (arrow). **c**, Metastatic tumor, in which the immunostaining is greater in all regions, and can also be observed in the cytoplasm of crypt cells (arrows). Magnification 40X

The existence of alterations in the expression of genes involved in both the polymerization and the modification of the CS chains prompted us to conduct an immunohistochemical analysis of the structure of these saccharide chains using the specific antibody CS-56. Healthy tissues displayed most staining in the stroma, while staining intensity increased in tumors, where it was associated with the fibrous elements of the matrix in non-metastatic LSCRCs, as well as the cytoplasm of some cells (Fig. 10).

### Relationship between alteration in gene expression and survival data

Of the genes that showed statistical differences in their expression patterns, we found better survival medians in those patients with overexpression of *GPC4* and *HS6ST3*, and underexpression of *GPC6*, *PRCAN*, *NDST1*, *HS6ST1*, *CHST11*, *CHST3*, *DSE* and *UST*, although statistically better OS was only related to the underexpression of *GPC6* (68.10 vs. 37.33 months, *p* = 0.077), *NDST1* (68.10 vs. 50.00 months, *p* = 0.035), *HS6ST1* (79.47 vs. 51.40 months, *p* = 0.068) and *CHST12* (68.10 vs. 12.30 months, *p* = 0.026) (Additional file 2: Table S2, and Additional file 3: Figure S3). This behavior persisted irrespective of lymph node involvement, especially when there was underexpression of *NDST1* (pN0 57.67 vs 12.30, pN1 68.10 vs. 50.00 months, *p* = 0.047) and *CHST12* (pN0 57.67 vs. 9.13 months, pN1 68.10 vs. 37.33 months, *p* = 0.042), and there was also a trend for the downregulation of *GPC6* (N0 57.67 vs. 9.13 months, pN1 68.10 vs. 37.33 months, *p* = 0.076) and *HS6ST1* (pN0 57.67 vs. 9.13 months, pN1 68.10 vs. 37.33 months, p = 0.042). On the other hand, overexpression of *SDC1*, *CHPF*, and *HS2ST1*, and downregulation of *COL18A1*, *SRGN* and *CSGALNACT2* were linked to poorer outcomes, though the effect was not statistically significant. No differences in OS were found for the expression of *NDST2*, *GLCE*, *HS3ST6*, and *CHST7*.

## Discussion

The abnormal expression of HSPGs in cancer and stromal cells can serve as a biomarker for tumor progression and patient survival [25]. HS fine structure is determined by the cell-type specific expression of only certain isoforms of some of the biosynthetic enzymes, notwithstanding the existence in some specific cases of regulation at the level of translation or enzymatic catalysis [28–30]. In a previous work, we have described the alterations that take place in RSCRCs which affect both the core proteins of HSPGs and the different enzymes responsible for the synthesis of the GAG chains, as well as the differences in these changes depending on the presence or not of metastasis in malignances [21]. In the present study, we provide a similar analysis focused on LSCRCs in order to determine whether there are any differences between left- and right sided pathologies. As in the previous study, here we have considered the comparative analysis of tumors at the T3 stage, where the muscularis propria is affected, and classified the tumors depending on the presence or absence of lymph node metastasis.

Two gene families, syndecans and glypicans, account for most cell surface HSPGs in humans, along with a few part-time proteins [22]. Transcripts for all syndecan species were detected in LSCRCs, but only syndecan-1 mRNA appeared overexpressed in most tumors, independent of the presence of metastasis. Although some previous studies using colon carcinoma cells have described alterations in the transcriptions of syndecan-2 and -4 [16], our previous work on RSCRCs was only able to detect overexpression of syndecan-1 in metastatic tumors [21]. Interestingly, in this work the analysis of the expression of syndecan-1 protein using immunohistochemistry provided the noteworthy finding that non-metastatic LSCRCs displayed lower immunoreactivity to that detected in normal tissues from the same patients, while the metastatic tumors showed a dramatic decrease in staining. Furthermore, non-metastatic tumors have a certain level of staining in the extracellular matrix, suggesting the shedding of cell membrane-bound proteoglycans. These results are very similar to those previously described in RSCRCs, where we suggested that the expression of syndecan-1 involves additional post-transcriptional mechanisms, such as protein translation, degradation, inhibition by feedback loops or miRNA regulation [21]. There is evidence for the post-transcriptional regulation of syndecan-1 expression in, for example, pancreatic cancer and peritoneal macrophages [30, 31]. Our data also correlate well with other previous immunohistochemical studies which have described a loss of expression of syndecan-1 in CRCs, some of which have been found to correlate with tumor stage and metastasis [32–35]. Upregulation of syndecan-1 has been described in some types of tumors, and it has been postulated that this aberrant expression may play a key role in promoting growth factor signaling in cancer cells [36]. In contrast, other malignances have been found to show downregulation of this molecule, indicating that this HSPG could well serve as a prognostic marker in a cancer-type-specific manner [36].

The glypican family comprises six cell surface HSPGs that are involved in the regulation of several signaling pathways, where, depending on biological context, they either stimulate or inhibit activity [37]. As such, tumor progression is affected by their activity, with abnormal expression being linked to various human tumors [25]. In the samples analyzed in this study, their relative expression patterns were quite similar to those observed in RSCRCs [21]. However, very few transcriptional changes were detected: in isoforms 4 and 6 in metastatic tumors, where, moreover, these alterations were markedly different from those observed in ascending tumors, where there is a great underexpression of glypican 1 in all types of tumors; and in isoforms − 3 and − 6 in non-metastatic tumors [21]. Unlike the other isoforms, relatively little is known concerning the expression or functional roles of glypican-4 and -6 in tumors. However, the ability of glypican-4 to uncouple pluripotent stem cell differentiation from tumorigenic potential has been recorded [38], while the reduced expression or loss of function of glypican-6 has been described in retinoblastoma and autosomal-recessive omodysplasia [39, 40].

Betaglycan and CD44v3 are part time membrane HSPGs, meaning that they occur either with or without HS chains [22]. Although the expression of CD44v3 in CRCs has been described as being related to more advanced pathological stage and poorer prognosis [41], in this study no statistically significant differences in any type of LSCRCs were found, mirroring our previous findings for RSCRCs [21]. The other part time HSPG analyzed was betaglycan, whose expression in tumor cells appears to play an important role in the progression of the pathology [42]. However, in relation to CRCs, although this molecule appeared underexpressed in non-metastatic RSCRCs [21], in this study no significant differences between tumor and healthy tissues was detected in LSCRCs.

Another cell-associated HSPG is serglycin, which constitutes a separate category since it has the peculiarity of being located intracellularly [23]. Transcript levels of this gene in this work were significantly reduced, both in metastatic and non-metastatic tumors, following a similar pattern to that previously observed in RSCRCs [21]. Serglycin is mainly found in hematopoietic and endothelial cells, and the principal GAG chains found bound to this core protein are CS, except in mast cells where CS type E or heparin may be present, depending on the cell’s origin [23, 43]. Mast cells in LSCRCs were drastically reduced in tumors compared to non-tumor colon mucosa, which could be, at least in part, the reason for the decrease in protein expression. A number of previous studies have described alterations in serglycin in different tumors [44–46], and it is also worth noting that results analogous to those described in this work, involving both downregulation of transcription and reduction in the population of mast cells, have also been obtained in RSCRCs [21], suggesting that this is a common feature of both CRC types.

Three HSPG species are located at the ECM: agrin, perlecan and collagen XVIII, and the latter two showed significant downregulation in tumoral samples, which is interesting considering that these two species also appeared modified in RSCRCs [21]. Perlecan expression, both at the transcription and the protein level, was diminished in tumors, independent of the presence or absence of lymph node metastasis. Perlecan, a critical regulator of growth factor-mediated signaling and angiogenesis, is an essential element in maintaining basement membrane homeostasis [47], likely indicating that it has a role to play in the progression of CRCs.

Collagen XVIII appeared downregulated to a statistically significant extent only in non-metastatic LSCRCs, while in RSCRCs its expression was significantly reduced in both metastatic and non-metastatic tumors [21]. However, decreases of about 50% were found in 70% of metastatic LSCRCs, a result that approached significance (*p* = 0.07), leading us to suggest that the results observed could be dependent on the individual sample analyzed, and that the real effect might occur similarly in all CRCs, regardless of their location. Several reports in other malignances describe different types of alterations in collagen XVIII depending on type of tumor, e.g., its expression increases in ovarian and pancreatic cancer, while it diminishes in liver and oral cancer [25].

In summary the patterns of alterations in the levels of expression of HSPG core proteins in CRCs is quite similar for ECM molecules, syndecans and serglycin, independent of tumor location, while glypicans display differences between RS- and LS- malignances.

The tissue-specific expression of individual HSPGs will determine when and where HS chains are expressed. For GAG chain generation, it is necessary to regulate the activity of many different enzymes, mainly GTs, located in the lumen of the Golgi apparatus [10]. The initial step in the biosynthesis of the chains is the creation of a tetrasaccharide linkage region, followed by polymerization through the consecutive addition of alternating GlcA and GlcNAc. A number of works have described variations in HS levels, both increases and decreases, in different tumor types [15, 48], including for CRCs, where decreases have been reported [17, 49]. However, in this work, it was not possible to determine the existence of significant differences in the transcription levels of any of these genes in LSCRCs. This finding contrasts with the results previously described for metastatic RSCRCs, in which *B3GAT1* expression decreased, particularly in non-metastatic RSCRCs, where several genes responsible for the synthesis of the linker (*XYLT1*, *XYLT2*, *B3GAT1*) and the polymerization of the chain (*EXT1*) were downregulated [21].

During HS biosynthesis, various sulfation and epimerization reactions take place which are responsible for the fine structure of the saccharide chain. The first reaction involved in polymer modification is the removal of acetyl groups from GlcNAc residues, after which the amino group is sulfated, catalyzed by four different isoforms of N-deacetylase/N-sulfotransferases [10]. The tissue distribution of NDST1 and NDST2 broadly overlap [50], and transcripts for both were detected in all samples analyzed in this study, with *NDST2* appearing downregulated in all LSCRCs, while *NDST1* transcription was downregulated in all metastatic tumors and in 60% of non-metastatic. In contrast, previous work with RSCRCs has shown that both isoforms were underexpressed, but only in the non-metastatic patients [21]. *NDST4* was undetectable in most samples in the current work, while *NDST3* transcripts were detected in only a small percentage of tumors. Expression of NDST3 and NDST4 is principally restricted to the period of embryonic development [51]. That said, in certain tumor types, expression of these molecules has been described, for example, NDST4 in breast cancer [51], although in RSCRCs neither was detected [21].

The next steps in the synthesis of the HS chains include the epimerization of GlcA into IdoA, an action catalyzed by the enzyme C5-GlcA epimerase, along with O-sulfation at C2 of uronic acid [11, 12]. An overexpression in the transcription levels of the two genes involved, *GLCE* and *HS2ST1*, was detected in non-metastatic LSCRCs in this study, although not in metastatic tumors, in contrast to our previous study in RSCRCs which found no alterations in the expression of these genes [21].

The addition of an O-sulfate group at C6 is mediated by enzymes encoded by the genes *HS6ST1–3*, each of which is specific to a particular substrate and differs in its tissue expression [52]. Transcripts for the three isoforms were identified in the healthy tissue studied here, but *HS6ST3* mRNAs were not detected in tumor samples, neither metastatic nor non-metastatic. Meanwhile, *HS6ST1* appeared downregulated in metastatic LSCRCs. These results show a pattern different from that previously described in RSCRCs, where *HS6ST2* was not detected in either tumor type or in healthy tissue, and *HS6ST1* appeared deregulated in non-metastatic tumors [21].

The last, and largest, family of enzymes involved in the biosynthesis of HS is the 3-O-sulfotransferases, which comprises seven different members (HS3ST1–6). In LSCRCs, only isoform − 6 expression appeared altered, its expression being diminished in non-metastatic tumors, although it was not detectable in metastatic forms. Again the data differ from those obtained in RSCRCs, where none of the isoforms appeared modified in metastatic tumors, and *HS3STB1* and *HS3ST5* were underexpressed only in non-metastatic ones [21]. 3-O-sulfation is a relatively rare modification, and it has only been found to influence a small number of proteins thus far [53]. That said, several studies have reported 3-O-sulfation alterations in different tumors [15, 48], and it has been suggested that certain patterns of 3-O-sulfation may be responsible for the appearance of cancerous phenotypics [25].

Once the HS biosynthetic process has been completed, 6S groups present at the glucosamine residues can be post-synthetically edited from the chain, suggesting that it may have special regulatory importance. The reaction is carried out by two endosulfatases located on the cell surface, SULF1 and SULF2 [27], and alterations in the expression of these genes in various tumor types, either up- or downregulation, have been reported [15, 48]. In the case of LSCRCs, transcript levels of none of these genes were found to be altered, mirroring the results previously observed in RSCRCs, where there were also no alterations [21].

Analysis of the HS structure by immunohistochemistry showed differences between normal mucosa and tumors as regards the intensity and distribution of the molecules. It is possible that these differences are caused by structural changes brought about by alterations in the transcription levels of HS biosynthetic enzymes.

Some of the HSPGs analyzed in this study are hybrid molecules, with both HS and CS side chains [54], making it interesting to extend the study to the genes involved in the biosynthesis of this GAG. In addition, changes in CSPGs associated with CRCs, such as versican and decorin, have been found [55], as well as alterations of the CS chains in RSCRCs [21].

CS chain extension takes place through the sequential addition of alternative GlcA and GalNAc residues. Five genes, *CSGALNACT1, CSGALNACT2*, *CHSY1*, *CHPF* and *CHSY3* encode the GTs involved in this process [56]. *CSGALNACT2* transcription was downregulated in non-metastatic LSCRCs, which coincides with what has been described in RSCRCs, although in the latter the rest of the genes involved, except *CHPF*, also experienced a decrease in their transcription levels [21]. Interestingly, *CHPF* appeared overexpressed in all LSCRCs, as it was in metastatic RSCRCs [21]. In other previous studies of CRCs, different changes have been described, although it must be stressed that these studies involved colorectal cancers from different locations and not only RSCRCs [57].

CS repeating disaccharide building units can be modified by epimerization of GlcA residues and by various sulfations [57]. O-sulfation at C4 of GalNAc residues is carried out by enzymes encoded by four genes (*CHST11–14*), although in this work only *CHST11* and − *12* appeared underexpressed in all LSCRCs. This concurs with our previous study in RSCRCs that showed a very similar expression pattern for these genes, although in that case *CHST14* was also underexpressed in all tumors, irrespective of their metastatic features [21].

O-sulfation at C6 of GalNAc residues is performed by three different genes, and *CHST3* translation was downregulated in non-metastatic tumors, although immunohistochemistry showed the opposite result, i.e. that it was upregulated. This apparent contradiction between the two sets of results is similar to the case described above for syndecan 1, and suggests the involvement of additional post-transcriptional mechanisms [21]. Discordances between mRNA and protein in complex biological samples have been widely analyzed and discussed [58], and subsets of proteins displaying negative correlation with mRNA expression values have been described in some tumors [59]. In addition, *CHST7* appeared underexpressed in metastatic LSCRCs in this work. Although very similar patterns of expression for the three genes were previously found in RSCRCs, no alterations in transcription were observed [21].

Both genes encoding the enzymes involved in the modification reactions of uronic acid residues, *DSE* and *UST*, were significantly altered, although DSE to a lesser extent, with the difference not reaching significance in metastatic tumors. The alterations observed once again followed a pattern similar to that previously observed in the ascending tumors, where both enzymes appeared downregulated [21]. The alterations in the CS chains as a result of the differences in expression of biosynthetic genes were analyzed by immunohistochemistry using the specific antibody CS-56, with clear differences found in the amount and location of the staining, although it must be taken into account that CS-56 antibody reacts preferentially with CS-D (sulfated at C-2 and C-6), but is also able to recognize other types of structures, including CS-A, -B, -C, and -E [60].

In terms of survival, the small sample size and the retrospective character of this study should be recognized as a considerable limitation, but, despite this, statistically significant differences in the underexpression of two genes were detected, along with a trend in two additional genes, and this behavior seems to be maintained regardless of lymph node involvement or not. Some of the other genes found to be significantly dysregulated in this work might also show a relationship with survival in a bigger sample, therefore prospective studies with a larger study population are necessary.

## Conclusions

In sum, analyzing the differential expression of the genes involved in HSPG biosynthesis in LSCRCs has highlighted that some of them showed significant changes in their transcript levels. When comparing the results with those previously described for ascending tumors, similarities can be seen for different gene families, as well as notable differences that evidence the heterogeneous character of the two pathologies. In contrast to RSCRCs, where non-metastatic tumors showed more alterations than metastatic tumors, in the case of LSCRCs the percentage was very similar, around 30%. With regard to HSPG core proteins, the patterns of alterations in expression of ECM molecules, syndecans and serglycin were quite similar, while glypicans displayed differences between RS- and LS- malignances.

The synthesis of the HS chains did not show, in contrast to RSCRCs, differences in the expression of the genes responsible for polymerization, but differences were evident in those controlling sulfation and epimerization. Differences between LS- and RSCRCs were preferentially found in the expression of genes involved in C6 and C3 sulfation of glucosamine, but not in NDSTs or SULFs. Finally, the synthesis of the CS chains showed differences with respect to the RSCRCs which preferentially affected the polymerization of the chains and the 6C sulfation of the GalNAc residue. The differences described in this article can help to understand some of the molecular differences between the proximal and distal colorectum previously described for various biomarkers.

## Additional files

Additional file 1: **Table S1.** Antibodies and dilution used. (PDF 11 kb)

Additional file 2: **Table S2.** Overall survival analysis. (PDF 29 kb)

Additional file 3: **Figure S3.** Overall survival in genes displaying statistically significant differences. Only genes with statistical differences are included in this figure. NDST1 underexpression provided a better outcome with an 18-month benefit. The magnitude of the benefit was even higher when CHST12 was downregulated, in more than 50-month. This difference was maintained regardless of lymph node involvement. GCP6 and HS6ST1 underexpression also presented better outcomes, but this magnitude did not reach statistical difference when stratified by node status. (PDF 260 kb)

## Abbreviations

CRC: Colorectal cancer
CS: Chondroitin sulfate
ECM: Extracellular matrix
GAG: Glycosaminoglycan
GalNAc: N-acetyl-D-galactosamine
GlcA: D-glucuronic acid
GlcNAc: N-acetyl-D-glucosamine
GT: Glycosyltransferase
HS: Heparan sulfate
HSPG: Heparan sulfate proteoglycan
IdoA: Iduronate
LSCRC: Left sided colorectal cancer
OS: Overall survival
qRT-PCR: Quantitative real-time polymerase chain reaction
RSCRC: Right sided tumors

## References

[1] Manne U, Shanmugam C, Katkoori VR, Bumpers HL, Grizzle WE (2010). Development and progression of colorectal neoplasia. Cancer Biomark.

[2] Chau BN, Diaz RL, Saunders MA, Cheng C, Chang AN, Warrener P (2009). Identification of SULF2 as a novel transcriptional target of p53 by use of integrated genomic analyses. Cancer Res.

[3] Baraz L, Haupt Y, Elkin M, Peretz T, Vlodavsky I (2006). Tumor suppressor p53 regulates heparanase gene expression. Oncogene.

[4] Truant S, Bruyneel E, Gouyer V, De Wever O, Pruvot FR (2003). Requirement of both mucins and proteoglycans in cell-cell dissociation and invasiveness of colon carcinoma HT-29 cells. Int J Cancer.

[5] Lai JP, Oseini AM, Moser CD, Yu C, Elsawa SF, Hu C (2010). The oncogenic effect of sulfatase 2 in human hepatocellular carcinoma is mediated in part by glypican 3-dependent Wnt activation. Hepatology.

[6] Sakane H, Yamamoto H, Matsumoto S, Sato A, Kikuchi A (2012). Localization of glypican-4 in different membrane microdomains is involved in the regulation of Wnt signaling. J Cell Sci.

[7] Zhao W, McCallum SA, Xiao Z, Zhang F, Linhardt RJ (2012). Binding affinities of vascular endothelial growth factor (VEGF) for heparin-derived oligosaccharides. Biosci Rep.

[8] Mahtouk K, Cremer FW, Rème T, Jourdan M, Baudard M, Moreaux J (2006). Heparan sulphate proteoglycans are essential for the myeloma cell growth activity of EGF-family ligands in multiple myeloma. Oncogene.

[9] Rider CC (2006). Heparin/heparan sulphate binding in the TGF-beta cytokine superfamily. Biochem Soc Trans.

[10] Esko JD, Lindahl U (2001). Molecular diversity of heparan sulphate. J Clin Invest.

[11] Whitelock JM, Iozzo RV (2005). Heparan sulfate: a complex polymer charged with biological activity. Chem Rev.

[12] Park PW, Reizes O, Bernfields M (2000). Cell surface heparan sulfate proteoglycans: selective regulators of ligand-receptor encounters. J Biol Chem.

[13] Sanderson RD (2001). Heparan sulfate proteoglycans in invasion and metastasis. Semin Cell Dev Biol.

[14] Lindahl U, Kjellén L (2013). Pathophysiology of heparan sulphate: many diseases. few drugs J Intern Med.

[15] García-Suárez O, Fernández-Vega I, Quirós LM (2013). Multiple alterations of heparan sulfate in cancer. OA Cancer.

[16] Park H, Kim Y, Lim Y, Han I, Oh ES (2002). Syndecan-2 mediates adhesion and proliferation of colon carcinoma cells. J Biol Chem.

[17] Bouziges F, Simon-Assmann P, Leberquier C, Marescaux J, Bellocq JP, Haffen K (1990). Changes in glycosaminoglycan synthesis and in heparan sulfate deposition in human colorectal adenocarcinomas. Int J Cancer.

[18] Jayson GC, Lyon M, Paraskeva C, Turnbull JE, Deakin JA, Gallagher JT (1998). Heparan sulfate undergoes specific structural changes during the progression from human colon adenoma to carcinoma in vitro. J Biol Chem.

[19] Karibe T, Fukui H, Sekikawa A, Shiratori K, Fujimori T (2008). EXTL3 promoter methylation down-regulates EXTL3 and heparan sulphate expression in mucinous colorectal cancers. J Pathol.

[20] Miyamoto K, Asada K, Fukutomi T, Okochi E, Yagi Y, Hasegawa T (2003). Methylation-associated silencing of heparan sulfate D-glucosaminyl 3-O-sulfotransferase-2 [3-OST-2] in human breast, colon, lung and pancreatic cancers. Oncogene.

[21] Fernández-Vega I, García-Suárez O, García B, Crespo A, Astudillo A, Quirós LM (2015). Heparan sulfate proteoglycans undergo differential expression alterations in right sided colorectal cancer, depending on their metastatic character. BMC Cancer.

[22] Iozzo RV, Schaefer L (2015). Proteoglycan form and function: a comprehensive nomenclature of proteoglycans. Matrix Biol.

[23] Kolset SO, Tveit H (2008). Serglycin--structure and biology. Cell Mol Life Sci.

[24] Mikami T, Kitagawa H (2013). Biosynthesis and function of chondroitin sulfate. Biochim Biophys Acta.

[25] Theocharis A, Skandalis SS, Tzanakakis GN, Karamanos NK (2010). Proteoglycans in health and disease: novel roles for proteoglycans in malignancy and their pharmacological targeting. FEBS J.

[26] Nairn AV, Kinoshita-Toyoda A, Toyoda H, Xie J, Harris K, Dalton S (2007). Glycomics of proteoglycan biosynthesis in murine embryonic stem cell differentiation. J Proteome Res.

[27] Kreuger J, Kjellén L (2012). Heparan sulfate biosynthesis: regulation and variability. J Histochem Cytochem.

[28] Arvatz G, Barash U, Nativ O, Ilan N, Vlodaavsky I (2010). Post-transcriptional regulation of heparanase gene expression by a 3’ AU-rich element. FASEB J.

[29] Grobe K, Esko JD (2002). Regulated translation of heparan sulfate N-acetylglucosamine N-deacetylase/N-sulfotransferase isozymes by structured 5′-untranslated regions and internal ribosome entry sites. J Biol Chem.

[30] Yeaman C, Rapraeger AC (1993). Post-transcriptional regulation of syndecan-1 expression by cAMP in peritoneal macrophages. J Cell Biol.

[31] Conejo JR, Kleeff J, Koliopanos A, Matsuda K, Zhu ZW, Goecke H (2000). Syndecan-1 expression is up-regulated in pancreatic but not in other gastrointestinal cancers. Int J Cancer.

[32] Day RM, Hao X, Ilyas M, Daszak P, Talbot IC, Forbes A (1999). Changes in the expression of syndecan-1 in the colorectal adenoma-carcinoma sequence. Virchows Arch.

[33] Peretti T, Waisberg J, Mader AM, de Matos LL, da Costa RB, Conceicao GM (2008). Heparanase-2, syndecan-1, and extracellular matrix remodeling in colorectal carcinoma. Eur J Gastroenterol Hepatol.

[34] Hashimoto Y, Skacel M, Adams JC (2008). Association of loss of epithelial syndecan-1 with stage and local metastasis of colorectal adenocarcinomas: an immunohistochemical study of clinically annotated tumors. BMC Cancer.

[35] Mitselou A, Skoufi U, Tsimogiannis KE, Briasoulis E, Vougiouklakis T, Arvanitis D (2012). Association of syndecan-1 with angiogenesis-related markers, extracellular matrix components, and clinicopathological features in colorectal carcinoma. Anticancer Res.

[36] Raman K, Kuberan B (2010). Chemical tumor biology of Heparan sulfate proteoglycans. Curr Chem Biol.

[37] Filmus J, Capurro M, Rast J (2008). Glypicans. Genome Biol.

[38] Fico A, De Chevigny A, Egea J, Bösl MR, Cremer H, Maina F (2012). Modulating Glypican4 suppresses tumorigenicity of embryonic stem cells while preserving self-renewal and pluripotency. Stem Cells.

[39] Lau CS, Yu CB, Wong HK, Fan DS, Wong KW, Lam DS (2010). Allelic imbalance at 13q31 is associated with reduced GPC6 in Chinese with sporadic retinoblastoma. Br J Ophthalmol.

[40] Campos-Xavier AB, Martinet D, Bateman J, Belluoccio D, Rowley L, Tan TY (2009). Mutations in the heparan-sulfate proteoglycan glypican 6 (GPC6) impair endochondral ossification and cause recessive omodysplasia. Am J Hum Genet.

[41] Kuniyasu H, Oue N, Tsutsumi M, Tahara E, Yasui W (2001). Heparan sulfate enhances invasion by human colon carcinoma cell lines through expression of CD44 variant exon 3. Clin Cancer Res.

[42] Bernabeu C, Lopez-Novoa JM, Quintanilla M (2009). The emerging role of TGF-beta superfamily coreceptors in cancer. Biochim Biophys Acta.

[43] Korpetinou A, Skandalis SS, Labropoulou VT, Smirlaki G, Noulas A, Karamanos NK (2014). Serglycin: at the crossroad of inflammation and malignancy. Front Oncol.

[44] Korpetinou A, Skandalis SS, Moustakas A, Happonen KE, Tveit H, Prydz K (2013). Serglycin is implicated in the promotion of aggressive phenotype of breast cancer cells. PLoS One.

[45] He L, Zhou X, Qu C, Tang Y, Zhang Q, Hong J (2013). Serglycin (SRGN) overexpression predicts poor prognosis in hepatocellular carcinoma patients. Med Oncol.

[46] Li XJ, Ong CK, Cao Y, Xiang YQ, Shao JY, Ooi A (2011). Serglycin is a theranostic target in nasopharyngeal carcinoma that promotes metastasis. Cancer Res.

[47] Iozzo RV, Zoeller JJ, Nyström A (2009). Basement membrane proteoglycans: modulators par excellence of cancer growth and angiogenesis. Mol Cells.

[48] Fernández-Vega I, García B, García-Suárez O, Castañón S, Quirós LM (2014). Alterations of Heparan sulfate proteoglycans in Cancer. J Glycobiol.

[49] Joo EJ, Weyers A, Li G, Gasimli L, Li L, Choi WJ (2014). Carbohydrate-containing molecules as potential biomarkers in Colon Cancer. OMICS.

[50] Grobe K, Ledin J, Ringvall M, Holmborn K, Forsberg E, Esko JD (2002). Heparan sulfate and development: differential roles of the N-acetylglucosamine N-deacetylase/N-sulfotransferase isozymes. Biochim Biophys Acta.

[51] Fernández-Vega I, García O, Crespo A, Castañón S, Menéndez P, Astudillo A (2013). Specific genes involved in synthesis and editing of heparan sulphate proteoglycans show altered expression patterns in breast cancer. BMC Cancer.

[52] Smeds E, Habuchi H, Do AT, Hjertson E, Grundberg H, Kimata K (2003). Substrate specificities of mouse heparan sulphate glucosaminyl 6-O-sulphotransferases. Biochem J.

[53] Thacker BE, Xu D, Lawrence R, Esko JD (2014). Heparan sulfate 3-O-sulfation: a rare modification in search of a function. Matrix boil.

[54] Iozzo RV (2001). Heparan sulfate proteoglycans: intrincate molecules with intriguing functions. J Clin Invest.

[55] Asimakopoulou AP, Theocharis AD, Tzanakakis GN, Karamanos NK (2008). The biological role of chondroitin sulfate in cancer and chondroitin-based anticancer agents. In Vivo.

[56] Mizumoto S, Ikegawa S, Sugahara K (2013). Human genetic disorders caused by mutations in genes encoding biosynthetic enzymes for sulfated glycosaminoglycans. J Biol Chem.

[57] Kalathas D, Theocharis DA, Bounias D (2011). Chondroitin synthases I, II, III and chondroitin sulfate glucuronyltransferase expression in colorectal cancer. Mol Med Rep.

[58] Maier T, Güell M, Serrano L (2009). Correlation of mRNA and protein in complex biological samples. FEBS Lett.

[59] Chen G, Gharib TG, Huang CC, Taylor JM, Misek DE, Kardia SL (2002). Discordant protein and mRNA expression in lung adenocarcinomas. Mol Cell Proteomics.

[60] Ito Y, Hikino M, Yajima Y, Mikami T, Sirko S, von Holst A (2005). Structural characterization of the epitopes of the monoclonal antibodies 473HD, CS-56, and MO-225 specific for chondroitin sulfate D-type using the oligosaccharide Library. Glycobiology.

