# Supplementary material for: Heparan sulfate proteoglycans undergo differential expression alterations in left sided colorectal cancer, depending on their metastatic character
Source: BMC Cancer. 2018 Jun 25;18:687. doi: 10.1186/s12885-018-4597-x (PMC6019305; doi:10.1186/s12885-018-4597-x)
Supplement: Supplementary file 1 — Table S1. Antibodies and dilution used. (PDF 11 kb) [file 12885_2018_4597_MOESM1_ESM.pdf]

Table S1. Antibodies and dilution used.

Antigen	Species of origin	Dilution	Supplier
**C6ST-1 (H-110)**	Rabbit	1:50	Santa Cruz Biotechnology
**CD117**	Rabbit	1:500	Dakocytomation
**ColXVIII**	Mouse	1:100	Thermoscientific
**CS (Clone CS-56)**	Mouse	1:100	Sigma-Aldrich Corp
**CS4ST2**	Goat	1:100	Santa Cruz Biotechnology
**Glypican -4**	Rabbit	1:100	Thermo Scientific
**HS (10E4 epitope)**	Mouse	1:50	amsbio
**HS2ST1 (Z-23)**	Rabbit	1: 100	Santa Cruz Biotechnology
**NDST1**	Mouse	1:50	Santa Cruz Biotechnology
**Perlecan**	Rabbit	1:100	Santa Cruz Biotechnology
**Sydecan1 (CD138)**	Mouse	1:25	Dakocytomation
**UST**	Rabbit	1:100	Thermoscientific
