# Supplementary material for: Heparan sulfate proteoglycans undergo differential expression alterations in left sided colorectal cancer, depending on their metastatic character
Source: BMC Cancer. 2018 Jun 25;18:687. doi: 10.1186/s12885-018-4597-x (PMC6019305; doi:10.1186/s12885-018-4597-x)
Supplement: Supplementary file 2 — Table S2. Overall survival analysis. (PDF 29 kb) [file 12885_2018_4597_MOESM2_ESM.pdf]

Table S2. Overall survival analysis

			Median OS	P value
**Global (all LCRC cases)**			57,670	
**Node Status**	pN0		51,400	NS
	pN1		62,870	
**GPC6 underexpression**	No		37,330	,077 *,076*
		pN0	*9,130*	
		pN1	*37,330*	
	Yes		68,100	
		pN0	*57,670*	
		pN1	*68,100*	
**NDST1 underexpression**	No		50,000	,035 *,047*
		pN0	*12,300*	
		pN1	*50,000*	
	Yes		68,100	
		pN0	*57,670*	
		pN1	*68,100*	
**HS6ST1 underexpression**	No		51,400	,068 *NS*
		pN0	*51,400*	
		pN1	*50,000*	
	Yes		79,470	
		pN0	.	
		pN1	*68,100*	
**CHST12 underexpression**	No		12,300	,026 *,042*
		pN0	*9,130*	
		pN1	*37,330*	
	Yes		68,100	
		pN0	*57,670*	
		pN1	*68,100*	

OS: Overall survival
NS: No statistical difference
