# Supplementary figures and images for: Heparan sulfate proteoglycans undergo differential expression alterations in left sided colorectal cancer, depending on their metastatic character
Source: BMC Cancer. 2018 Jun 25;18:687. doi: 10.1186/s12885-018-4597-x (PMC6019305; doi:10.1186/s12885-018-4597-x)

Figure S3. Overall survival in genes displaying statistically significant differences.

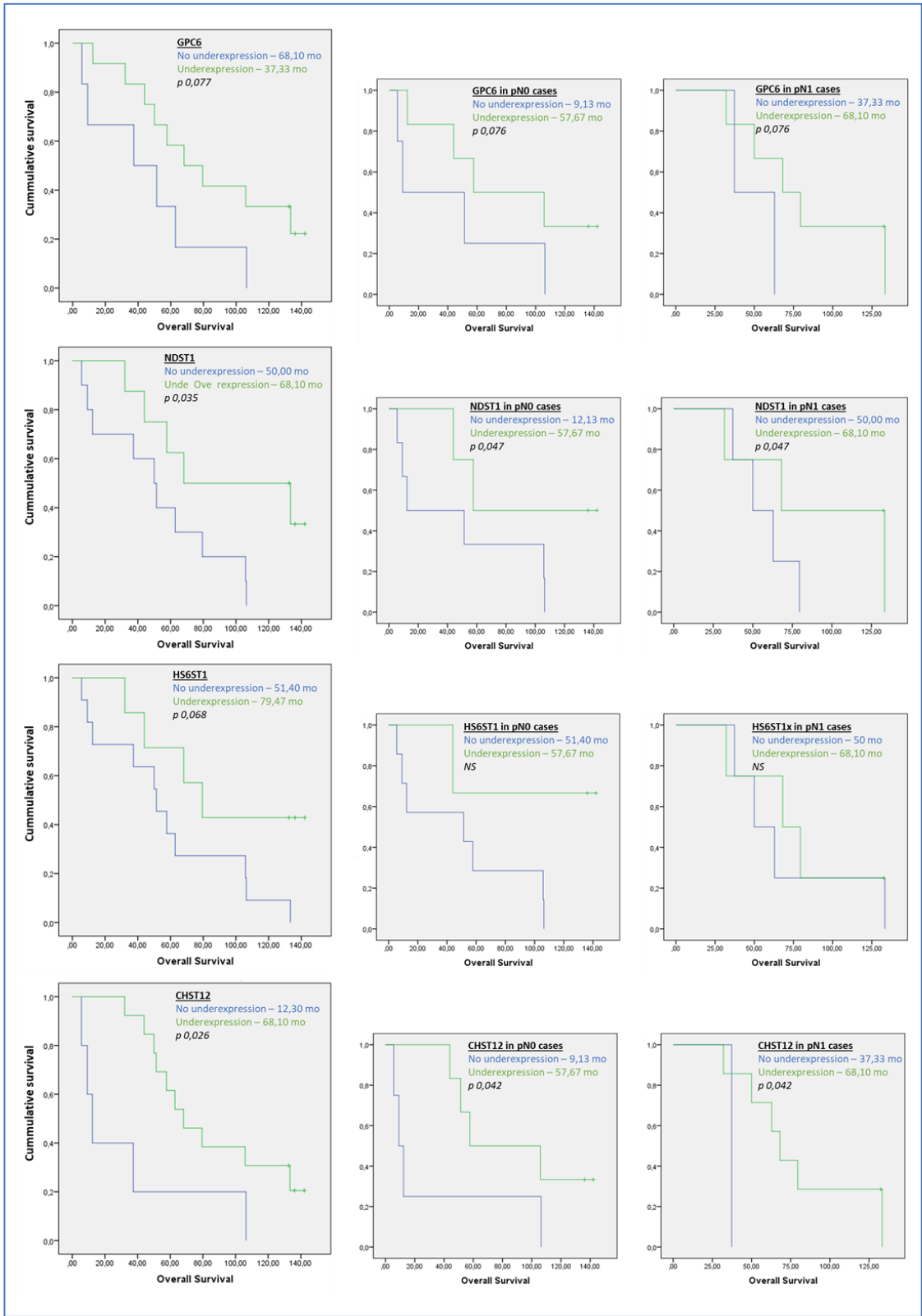

Supplement: Supplementary file 3 — Figure S3. Overall survival in genes displaying statistically significant differences. Only genes with statistical differences are included in this figure. NDST1 underexpression provided a better outcome with an 18-month benefit. The magnitude of the benefit was even higher when CHST12 was downregulated, in more than 50-month. This difference was maintained regardless of lymph node involvement. GCP6 and HS6ST1 underexpression also presented better outcomes, but this magnitude did not reach statistical difference when stratified by node status. (PDF 260 kb) [file 12885_2018_4597_MOESM3_ESM.pdf]
